# Sequential Broadening of CTL Responses in Early HIV-1 Infection Is Associated with Viral Escape

**DOI:** 10.1371/journal.pone.0000225

**Published:** 2007-02-21

**Authors:** Annika C. Karlsson, Astrid K.N. Iversen, Joan M. Chapman, Tulio de Oliveira, Gerald Spotts, Andrew J. McMichael, Miles P. Davenport, Frederick M. Hecht, Douglas F. Nixon

**Affiliations:** 1 Gladstone Institute of Virology and Immunology, University of California, San Francisco, California, United States of America; 2 The Swedish Institute for Infectious Disease Control, and Department of Microbiology, Tumor and Cell Biology, Karolinska Institutet, Solna, Sweden; 3 MRC Human Immunology Unit, Weatherall Institute of Molecular Medicine, Oxford, United Kingdom; 4 Department of Zoology, University of Oxford, Oxford, United Kingdom; 5 Division of Experimental Medicine, Department of Medicine, University of California, San Francisco, California, United States of America; 6 University of New South Wales, Sydney, Australia; New York University School of Medicine, United States of America

## Abstract

**Background:**

Antigen-specific CTL responses are thought to play a central role in containment of HIV-1 infection, but no consistent correlation has been found between the magnitude and/or breadth of response and viral load changes during disease progression.

**Methods and Findings:**

We undertook a detailed investigation of longitudinal CTL responses and HIV-1 evolution beginning with primary infection in 11 untreated HLA-A2 positive individuals. A subset of patients developed broad responses, which selected for consensus B epitope variants in Gag, Pol, and Nef, suggesting CTL-induced adaptation of HIV-1 at the population level. The patients who developed viral escape mutations and broad autologous CTL responses over time had a significantly higher increase in viral load during the first year of infection compared to those who did not develop viral escape mutations.

**Conclusions:**

A continuous dynamic development of CTL responses was associated with viral escape from temporarily effective immune responses. Our results suggest that broad CTL responses often represent footprints left by viral CTL escape rather than effective immune control, and help explain earlier findings that fail to show an association between breadth of CTL responses and viral load. Our results also demonstrate that CTL pressures help to maintain certain elements of consensus viral sequence, which likely represent viral escape from common HLA-restricted CTL responses. The ability of HIV to evolve to escape CTL responses restricted by a common HLA type highlights the challenges posed to development of an effective CTL-based vaccine.

## Introduction

HIV-1-specific CTLs have a central role in the containment of viral replication. Reduction in viral load during acute HIV-1 infection is correlated with the appearance of HIV-specific CTLs [1], [2], and studies from the SIV macaque model have shown that CD8-specific monoclonal antibodies, which block CTL activity, can eliminate this early viral load reduction [3], [4], [5]. Also, in monkeys who received experimental SIV vaccines and subsequently were infected with pathogenic SIV, the frequency of mutations within CTL epitopes was associated with the level of viral replication [6].

Many studies have tried to correlate the magnitude and/or breadth of the CTL response with control of viremia in humans and for the majority of HLA restriction elements no such simple relationship exists. In chronic HIV-1 infection both broad and narrow high frequency CTL responses have been seen in patients whose CD4+ T cell counts are rapidly declining, or who are at late stage disease, as well as in asymptomatic patients with stable CD4+ T cell counts and low levels of viremia [7], [8], [9], [10], [11]. Still, immunogenetic studies have clearly shown that certain HLA alleles, e.g. HLA-B27, -B57 and –B58, are associated with long-term non-progression (LTNP), while HLA-B35 is associated with rapid disease progression [11], [12], [13], [14], [15], [16], [17], underscoring that the efficiency of viral containment varies with CTL specificity.

HIV-1 has been shown to escape host CTL responses through mutations in CTL epitopes in early infection as well as throughout the chronic phase of infection [18], [19], [20], [21], [22]. The speed at which a CTL response induces escape is thought to be dependent on several factors including the potency of the CTL response, how many nucleotides have to change to create an amino acid mutation, the effectiveness of the mutation in escaping CTL responses, and how well the change is accepted structurally and functionally (i.e. impact on viral fitness). Thus, a CTL response directed towards an epitope does not always lead to escape [23], [24], [25]. Of particular interest is the finding that delayed emergence of CTL escape mutants within an immunodominant HLA-B27-restricted epitope in Gag can lead to an increase in viral load and outgrowth of virus carrying escape mutations [7], [8], [26]. The CTL selection pressure exerted by HLA-B27 may not be greater than that exerted by other HLA restriction elements early in disease. Nevertheless, the protective effect of a delayed emergence of the Gag B-27-restricted escape mutations along with the frequent occurrence of compensatory mutations, may be related to the finding that escape, if it does occur, is associated with a viral fitness cost greater than that related to escape mutations in most other epitopes [27]. An additional case report showed that a combination of CTL escape and lost HIV-1-specific CD4+ T cell help can precede viral breakthrough in early chronic infection [28]. Thus, an effective, but narrow immunodominant CTL response against an epitopic region can persist until late in the natural history of HIV-1 disease and appears to be associated with control of viral replication.

HIV-1 continuously adapts to HLA-restricted CTL responses at the population level resulting in “HLA footprints” [29], [30], [31], [32], [33]. Several sites have been associated with positive selection by certain HLA alleles, while others are negatively associated with a different set of common HLA alleles. These associations reflect previous adaptation of the virus to the human host. After transmission of a CTL escape variant to a new host who does not carry the restrictive HLA allele, some variants are maintained while others revert, suggesting that those which revert are associated with a decrease in viral fitness [21], [34], [35]. Thus, the accumulation of mutations within the HIV-1 genome is not infinite [21], [36], [37], [38]. The stability of an escape mutation combined with the rate at which mutations are induced has important implications for vaccine development. Although a substantial number of studies have described viral escape, they mostly rely on case reports, a very limited number of patients and/or sequences, or are cross-sectional studies. Little is known about the early hierarchy in targeting CTL epitopes and relative immunodominance, the timing of escape mutations, and how efficient novel CTL responses can be generated towards epitope variants in acutely infected humans. Still, it is essential to evaluate the specificity and characteristics of immune responses that are able to control viral replication for the development of preventive or therapeutic vaccination.

To address these questions we undertook a comprehensive longitudinal study of eleven untreated individuals who presented with primary HIV-1 infection. We observed the complex interactions between an evolving viral population and the development of autologous cellular immune responses against optimally defined HLA-A2-restricted epitopes situated in Gag, Pol, Env, and Nef. We also investigated the interactions between CTL responses restricted by different HLA alleles in overlapping and flanking epitopes in the p17 region of HIV-Gag and HIV-Nef. We provide clear phylogenetic and functional evidence of HIV-1 CTL escape within targeted epitopes in HIV-Gag, Nef, and Pol. In two independent examples we show that CTL responses directed at variant epitope sequences in HIV-Gag and –Pol caused selection of a consensus B-like sequence. We also show that the lack of responses to the HIV-Nef epitope in primary infection were associated with a pre-existing CTL escape variant that was probably present in the transmitted virus, suggesting adaptation of HIV-1 to HLA-A2-restricted CTL responses at a population level. Finally, our results identify a paradox in which broad CTL responses in early infection, when linked to escape mutations, were associated with an increased HIV-1 load. Although early HLA-restricted responses may have helped limit viral load, subsequent CTL escape effectively abrogated the effects of the CTL response. The development of new CTL responses resulted in broader responses as the earlier temporarily effective responses persisted as immunological footprints. This has important clinical implications for the manipulation of CTL responses by therapeutic immunization. Great care at the epitope specific level may be needed if selective manipulation of CTL responses is to be beneficial.

## Methods

### Subjects

Eleven untreated HLA-A2-positive subjects from the OPTIONS cohort at the Positive Health Program, University of California, San Francisco were identified for the study (Table 1). Criteria for primary and early HIV-1 infection in the cohort have been presented previously [39]. The male subjects were infected through homosexual contact with HIV-1 strains belonging to subtype B. Our inclusion criteria were: (i) HLA-A2-positive, (ii) treatment naïve throughout study period, (iii) longitudinal samples available for a minimum of one year, and (iv) viral loads >50 copies/ml. A control group of 12 HLA-A2-negative subjects from the cohort was also identified (Table 1). The study was approved by the UCSF Institutional Review Board, and all subjects provided written informed consent.

**Table 1:** HLA-A2-positive and HLA-A2-negative patient characteristics.

Patient	Sex	Week^A^	HLA-A allele		HLA-B allele		Viral Load (copies/ml)	CD4 (cells/mm^3^)	CD8 (cells/mm^3^)
**OP-177** B	Male	10	**02**	31	15	40	8,805	360	342
**OP-428** B	Male	15	01	**02**	08	08	2,340	1,260	1,092
**OP-454**	Male	10	01	**02**	51	57	151	528	560
**OP-474** C	Male	10	**02**	**02**	40	42	1,420	493	629
**OP-478** C	Male	12	**02**	24	44	51	265	594	1,122
**OP-488**	Male	13	**02**	24	27	50	2,249	748	1 078
**OP-506**	Male	11	**02**	03	08	35	151,107	286	494
**OP-539** C	Male	6	**02**	03	44	47	58,189	588	945
**OP-581**	Male	11	01	**02**	27	51	41,605	616	1,100
**OP-583**	Male	9	**02**	31	14	40	48,413	495	765
**OP-599** B	Male	14	**02**	**02**	13	51	1,451	558	738
**HLA-A2-Negative**									
**OP-138**	Female	29	03	32	27	35	16,460	585	442
**OP-430**	Male	38	03	03	44	51	36,854	240	1,776
**OP-443**	Male	10	01	29	35	81	18,218	714	1,176
**OP-470**	Male	20	11	68	35	40	33,893	462	420
**OP-565**	Male	25	01	24	08	50	159,750	465	825
**OP-626**	Male	10	03	30	35	49	59,035	888	768
**OP-683**	Male	10	26	66	35	41	80,599	528	672
**OP-700**	Male	26	01	11	07	40	32,902	551	931
**OP-722**	Male	30	03	11	07	40	124,070	522	1,080
**OP-745**	Male	10	11	24	13	48	36,254	560	1,140
**OP-791**	Male	10	01	24	40	57	4,973	570	665
**OP-842**	Male	3	03	25	40	55	6,876	560	496

A Estimated week from infection. ^B^Subject who developed broad HLA-A2 restricted responses. ^C^Subjects who developed narrow HLA-A2-restricted CTL responses against SL9, after primary infection.

PBMC and plasma samples were obtained from the HLA-A2-positive subjects approximately every 24-weeks from primary HIV-1 infection. An initial sample was available at an estimated median of 11 weeks (interquartile range: 10–13) from infection (Table 1), a timepoint after the initial two months in which HIV-1 infected subjects have been shown to reach a steady viral load [40]. The date of HIV-1 infection was estimated based on prior data on the median time from exposure to acute retroviral syndrome or an indeterminate HIV-1 antibody test [41], the mid-point between last negative and first positive HIV-1 antibody tests, or the level of a less sensitive HIV-1 antibody test if the optical density was between 0.5 and 1.0, a range in which there has been shown to be a linear relationship between the less sensitive antibody results and the days since seroconversion [42]. The first plasma sample was obtained after peak viremia, and the median plasma viral load was 2,300 copies/ml (interquartile range: 1,400 to 35,500 copies/ml).

### HIV-1 quantification, sequencing and cloning

Plasma HIV RNA levels were determined by Bayer's branched DNA technique (Bayer Diagnostics, Emeryville, California, USA). Plasma HIV RNA was extracted, reverse transcribed and amplified using both population based sequencing and cloning [43], [44]. Our primers were selected to amplify HIV-1 gag (HXB2 nt position 790–1431), pol (HXB2 nt position 2147–3462), or nef (HXB2 nt position 8774–9540) (Table S1).

In brief, viral RNA was isolated from plasma using Dynabeads® Oligo(dT)_25_ (Invitrogen Corporation, Dynal Biotech, Oslo, Norway) and HIV-1 genes were reverse transcribed and amplified using the gene specific primers as recommended by the manufacturers of the Titan One Tube RT-PCR kit (Roche Diagnostics GmbH, Roche Applied Science, Penzberg, Germany). Second-round PCR reactions were carried out using the Expanded High Fidelity enzyme blend (Roche Diagnostics GmbH).

To decrease the number of possible PCR-introduced errors and ensure a high input of virus strains into the PCR, several amplifications were done in parallel and were pooled afterward [45]. In samples with low viral load, several individual PCR amplicons were sequenced. The PCR product was purified using ExoSAP-IT® (USB Corporation, Cleaveland, OH, USA), sequenced directly or cloned (TOPO TA cloning kit; Invitrogen, Carlsbad, CA, USA). Plasmid DNA was purified using QIAprep Spin (QIAGEN, Valencia, CA, USA) and sequenced. Sequences were imported and manually edited using Sequencher software (Gene Codes Corporation, Ann Arbor, MI, USA).

### HLA-A2-restricted epitopes

A total of 20 HLA-A2-restricted epitopes in HIV-1 Gag, Pol, Env and Nef were identified in the Los Alamos HIV Molecular Immunology Compendium (Table 2) [46]. Furthermore, our sequencing data disclosed several autologous viral epitope variants in Gag, Pol (except RT 309-319) and Nef, and peptides corresponding to both the autologous and all consensus B epitope sequences [47] were synthesized.

**Table 2:** HLA-A2 restricted epitopes in HIV-Gag, -Env, -Nef and –Pol.

Name	Sequence (Consesnsus B)	Nucleotide position (HXB2)
Gag 77-85 (SL9)	SLYNTVATL	1017→1043
Gag 151-159	TLNAWVKVV	1239→1265
Gag 363-370	VLAEAMSQV	1872→1898
Gag 433-442	FLGKIWPSHK	2086→2115
Env 121-129	KLTPLCVTL	6583→6609
Env 192-200	RLISCNTSV	6796→6822
Env 311-320	RGPGRAFYTT	7153→7182
Env 813-822	SLLNATAIAV	8659→8688
Nef 83-91 (AL9)	AAVDLSHFL	9041→9067
Nef 137-145	LTFGWCFKL	9205→9231
Nef 180-189 (VL10)	VLVWRFDSRL	9331→9361
PR 45-54	KMIGGIGGFI	2384→2413
PR 76-84	LVGPTPVNI	2477→2503
RT 3-12	SPIETVPVKL	2555→2584
RT 33-41	ALVEICTEM	2645→2671
RT 108-118	VLDVGDAYFSV	2871→2903
RT 179-87 (VL9)	VIYQYMDDL	3083→3109
RT 181-189	YQYMDDLYV	3089→3115
RT 209-220	LLRWGLTTPDKK	3173→3208
RT 309-317 (IV9)	ILKEPVHGV	3473→3499

### Phylogenetic and selection analysis

The aligned sequences, obtained by direct sequencing and cloning, were analyzed together with reference sequences from the HIV sequence database (http://hiv-web.lanl.gov/) using MEGA to exclude the possibility of contamination. Viral variability and evolution were determined within the HLA-A2-restricted epitopes to reveal potential CTL escape (n = 15 epitopes). Mutations within epitopes or flanking regions were identified. The sequences have been submitted to GenBank (GenBank accession numbers: EF396480–EF396891).

Phylogenetic trees were constructed from sequenced clones obtained for patients OP177 (Nef), OP428 (Gag and Pol), and OP599 (Gag) by maximum likelihood using PAUP* 4.0b10 [48]. The GTR+Γ model of nucleotide substitution was used with model parameters estimated on an initial neighbour-joining tree. A heuristic search was then performed using SPR branch swapping. The substitution model parameters were re-estimated and the heuristic search repeated. Non-parametric bootstrap support estimates were obtained using 2000 replicates under neighbour-joining using the maximum likelihood substitution model.

Natural selection of specific amino acid sites in Gag, Nef and Pol were examined using the codeml program from the PAML package (version 3.13) [49]. This program works by assessing the fit of the data to various models of codon evolution while taking into account the phylogenetic relationships of the sequences. We used “NS-sites” models that allow the nonsynonymous/synonymous (dN/dS) ratio (ω) to vary among sites [50], [51] and examined six models: M0, considering one ratio for all sites; M1a, near neutrality (two classes of sites with ω<1 and  = 1); M2a, positive selection (three classes of sites ω<1,  = 1 and >1); M3, discrete ω categories; M7, discrete beta-distributed ratios; and M8, beta-distributed ratios with ω>1. Nested sets of these models [(M0, M1a, M2a, M3) and (M7, M8)] were examined using hierarchical likelihood ratio tests [52] and amino acid sites with Bayesian posterior dN/dS ratios reflecting positive selection were reported.

### Immunological measurements

The intracellular cytokine flow cytometry (CFC) assay was performed using frozen PBMC samples as previously described [53]. Briefly, cells were thawed, stimulated with peptides (2 µg/ml) and incubated overnight. The PBMC were stained with Pacific Blue (PB)-conjugated anti-CD4, and Phycoerythrin-Cy7 (PE-Cy7)-conjugated anti-CD8 (BD Biosciences, San Jose, CA, USA) for 20 minutes at 4°C. Simultaneous staining with 1 µg/ml ethidium monoazide bromide (EMA) was used to exclude dead cells. The cells were exposed to fluorescent light and fixed in 4% paraformaldehyde. The cells were permeabilized using FACS Perm solution (BD Biosciences), and stained using PE-Texas Red (ECD) conjugated CD3 (Beckman Coulter, Miami, FL, USA), fluorescein isothiocyanate (FITC)-conjugated anti-TNF-α, R-phycoerythrin (PE)-conjugated anti-IL-2, and allophycocyanin (APC)-conjugated anti-IFN-γ (BD Biosciences). The cells were fixed in 1% paraformaldehyde, and data was collected on a BD LSR-II using FACS DIVA software (BD Biosciences).

The gating strategy for all samples was to set a gate on the unstimulated control such that CD3+CD8+CD4-T cells were negative for IFN-γ and TNF-α. The background signal was a median of 0.07% (interquartile range: 0.03–0.18%) of the IFN-γ and TNF-α-producing CD8+ T cells and was subtracted in each experiment. A sample was considered positive (above the cutoff) when the response was at least twice the experimental background signal.

## Results

Previous studies of primary HIV-1 infection have shown limited responsiveness to several HLA-A2-restricted CTL epitopes [54], [55], [56], [57], even though responses against the HIV Gag 77-85 (SL9, ^77^SLYNTVATL^85^) epitope becomes immunodominant in chronic infection [55], [56], [58]. However, the timing of the onset of HLA-A2-restricted responses, their impact on viral evolution, and the potential efficiency of subsequent CTL responses generated towards autologous epitope variants are largely unknown. To address these questions, we identified eleven HLA-A2-positive patients in primary HIV-1 infection in the OPTIONS cohort according to the criteria described [39]; all had detectable viral loads (>50 copies/ml) and none received anti-retroviral therapy throughout the study period (Table 1). We furthermore included a control group of twelve drug-naïve, newly infected, HLA-A2-negative patients who also fulfilled the above viral load criteria (Table 1).

### Striking paucity of HLA-A2-restricted HIV-1 specific CTL responses at primary infection

**Figure 1:**
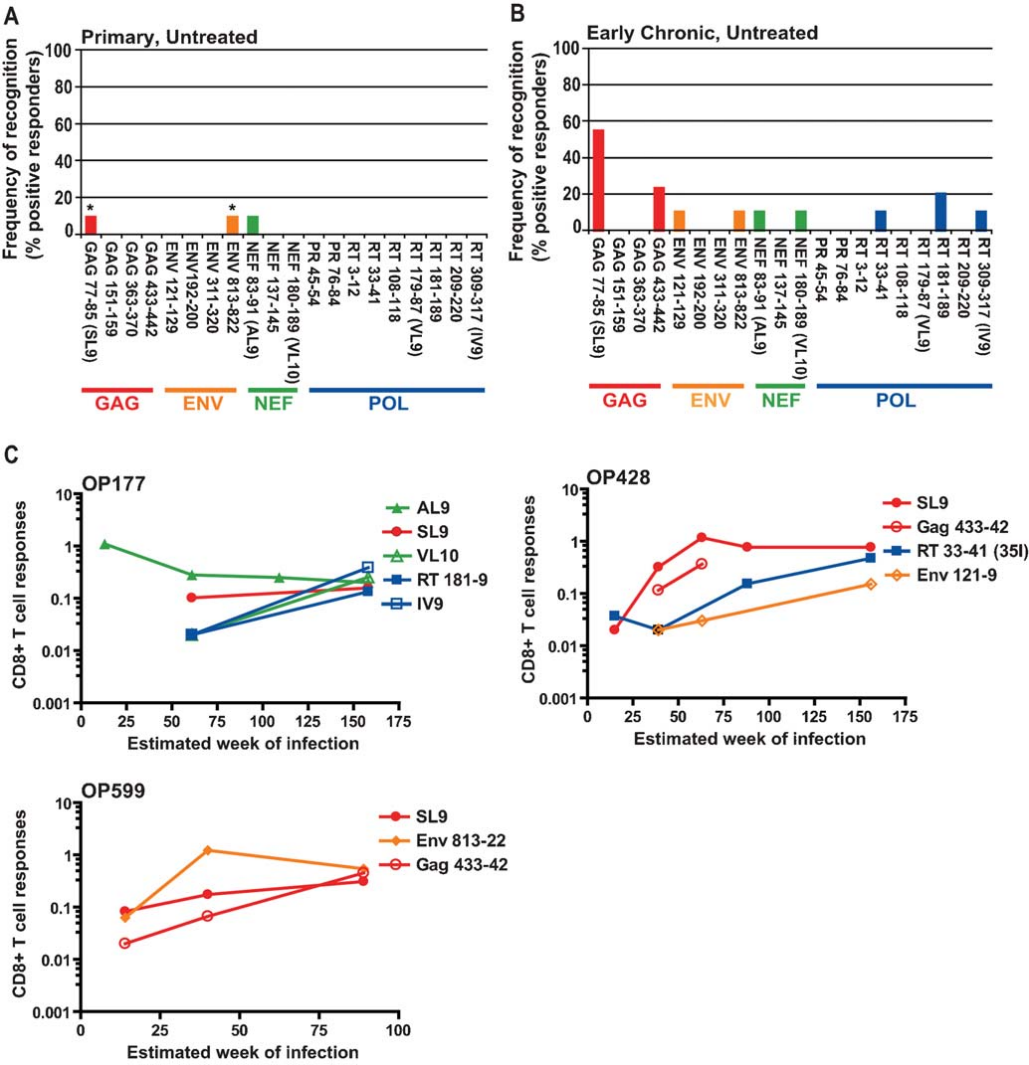
Continuous development of HLA-A2-restricted CTL responses. (A) At study entry only two of eleven patients (OP177 and OP599) showed detectable responses to three of the 20 HLA-A2-restricted epitopes tested. * Both epitopes were recognized by OP177. (B) At early chronic infection (week 48–144 of follow-up) most patients had developed a response against the Gag 77-85 (SL9) epitope. (C) Three of the eleven subjects (OP177, OP428, and OP599) developed broad HLA-A2-restricted CTL responses targeting four to five epitopes. The magnitude of the CD8+ T cell responses is given as the percentage of cells producing IFN-γ and TNF-α after withdrawing the experimental background.

We tested PBMC from our eleven HLA-A2-positive patients for secretion of IFN-gamma and TNF-alpha in the CFC assay using a panel of 20 HLA-A2-restricted peptides, which corresponded to autologous and consensus B epitope [46] variants from HIV-Gag, Pol, Env and Nef. At the first time-point tested, a median of 11 weeks (range 6 to 15 weeks) after HIV-1 infection, only two (OP599 and OP177) of the eleven patients were found to have HIV-specific HLA-A2 restricted CTL responses (Figure 1A). OP599 recognized two epitopes, one in Gag and the other in Env (SL9, and Env 813-22), and OP177 responded to one epitope in Nef (Nef 83-91, AL9), responses that showed up at week 14 and 13 post-infection, respectively for each patient. None of the targeted epitopes were located in HIV-Pol (protease and RT).

### Development of broad HLA-A2-restricted responses is associated with viral escape and an increased viral load during the first year of infection

**Figure 2:**
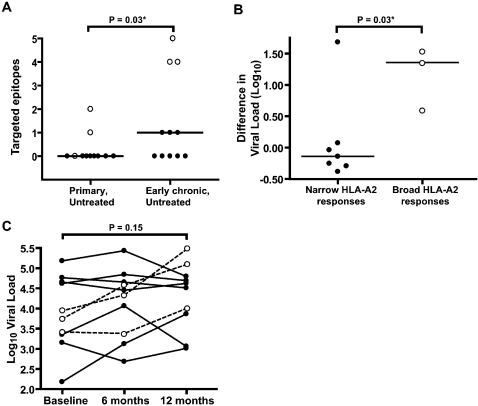
Broad HLA-A2-restricted CTL responses are associated with an early increase of viral load (A) The number of targeted epitopes in primary and early chronic infection increased significantly during the study-period (Paired T-test, p = 0.03). While only one patient recognized 2 epitopes in primary infection, three targeted at least 4 epitopes, situated in HIV-Gag, Env, Nef and Pol, in early chronic infection. (B) All three patients who targeted multiple epitopes (open circles) had a viral load increase >0.5 log_10_ during the first year of infection, compared to just one of seven patients with narrow or absent HLA-A2-restricted responses (closed circles) (Fisher's exact test, p = 0.03). Patient OP478, identified as being superinfected during the first year of infection, was excluded from this analysis. (C) While the viral load increased more in the group with broad HLA-A2-restricted responses during the first year of infection (open circles), the absolute viral load increase was not significant in the group during year one (Paired T-test, p = 0.15).

During the study period (range: 1–3 years; median: 2 years) there was a significant increase in the frequency of HLA-A2-restricted CTL responses in the subjects expressing HLA-A2 (Figure 2A, Paired T-test, p = 0.03). A total of 6/11 (55%) patients (Table 1) developed responses to one or more HIV-1 epitopes (Figure 1B, 2A). This response frequency is comparable to that found in chronic untreated patients (ACK, unpublished data). Two of these patients had HIV-1 specific CTL responses in primary infection and developed additional responses towards SL9, Nef 180-189 (VL10), RT 181-189, and RT 309-317 (IV9) (OP177) and Gag (p1) 433-442, and RT181-189 (OP599) (Figure 1C). Of the four patients who only developed responses after primary infection, all recognized the SL9 epitope in the HIV-Gag p17 region and one of these (OP428) also developed responses towards Gag (p1) 433-442, Env 121-129, and RT 33-41 (Figure 1C).

**Figure 3:**
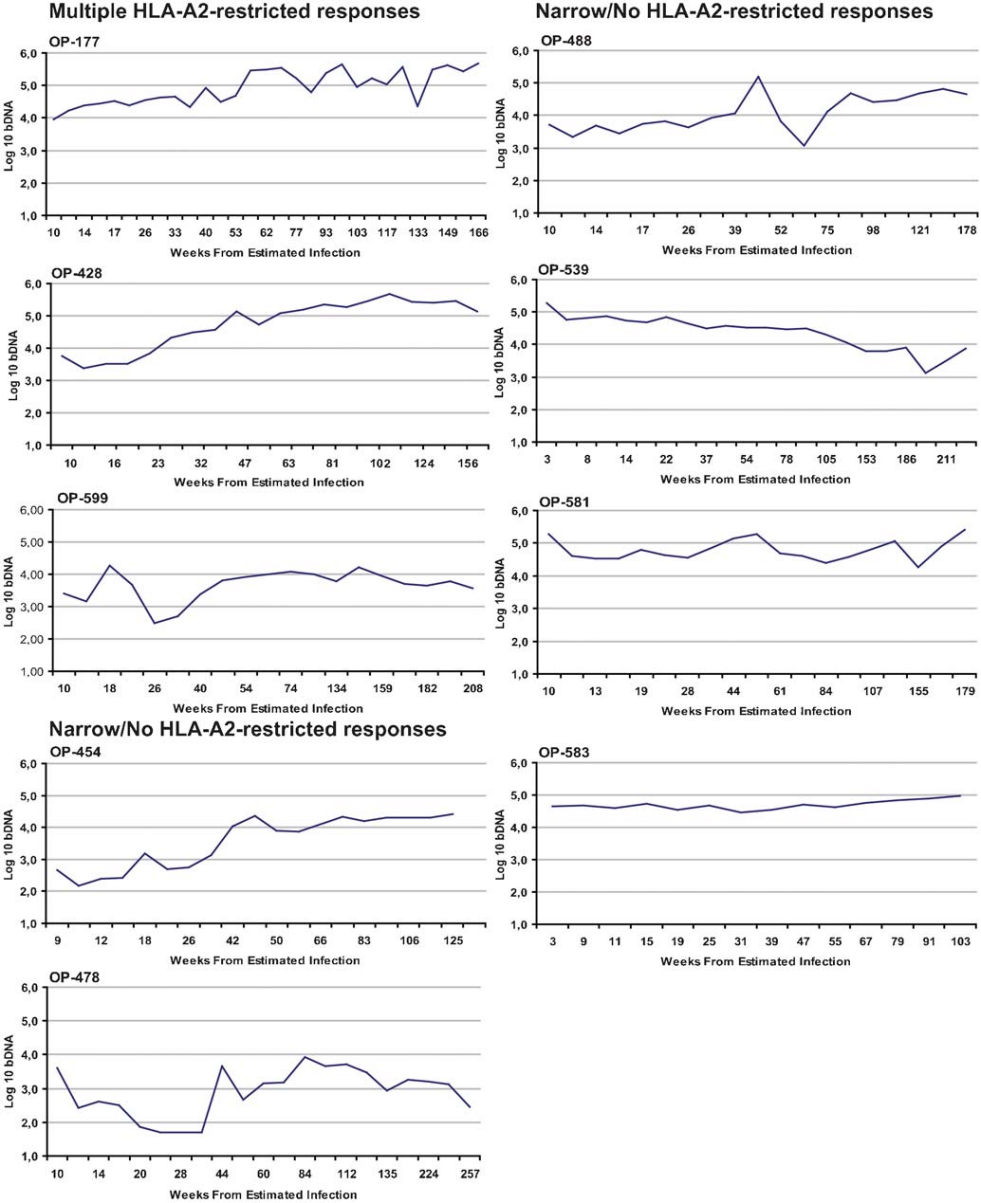
Longitudinal viral load measures in HLA-A2-positive subjects followed for more than one year (i.e. excluding OP474 and OP506). During the study period three subjects (OP177, OP428, and OP599) developed multiple HLA-A2-restricted CTL responses, which selected for mutations associated with viral escape within targeted epitopes. The additional six subjects followed for more than a year did not develop any HLA-A2-restricted responses or only against the SL9 epitope in HIV-Gag (OP478, and OP539).

Patients with broad CTL responses, OP177, OP428, and OP599, targeted four to five HLA-A2-restricted epitopes and our analysis showed that several of these epitopes over time developed mutations associated with CTL escape (described in detail below). This broadening of HLA-A2-restrcited responses during the study-period was associated with an increase in plasma viral load during the first year of infection (Figure 2B, 2C), as these three patients had a viral load increase of >0.5 log_10_, compared to only one of seven patients with no, or only SL9-specific, CTL responses (Fisher's exact test, p = 0.03). One HLA-A2-positive patient, OP478, identified as being superinfected with a second viral variant during the first year of infection (F.M.H., unpublished data) was excluded from this analysis. While the viral load increased more in the patients who developed broad CTL responses, at year one the average viral loads were similar (Figure 2C). Also, after year one the viral load remained stable for the duration of the study as shown in Figure 3. Thus the initial greater increase in viral loads did not lead to worse control of viral replication but to viral replication levels that were more similar to the rest of the group.

The limited reduction in viremia during year one in some of the subjects (Figure 2B) may reflect a slow ongoing reduction in viral density, under immunological control [40]. The outlier in this group, OP454, had a 1.7 log_10_ increase in viral load during the first year (151 to 7,261 copies/ml), had the lowest initial viral load of all patients (Figure 3), and did not develop any HLA-A2-restricted CTL responses during the three years studied. This patient was HLA-A1, B57, and B51 positive and may have had strong CTL responses restricted by any of these HLA molecules, undetected in our analysis. In a plasma sequence obtained at estimated week 106 from infection we did not detect any mutations within epitopes in HIV-Nef and HIV-Pol, but the I147L mutation located within the HLA-B57-restricted ^147^ISPRTLNAW^155^ (IW9) epitope in HIV-Gag was present, although not in association with the A146P mutation known to alter antigen processing [59]. In addition, the HIV-Gag sequence contained the G79F mutation located at the anchor position of the HLA-A1-restricted epitope ^71^GSEELRSLY^79^, which we show to have conferred viral escape in OP428.

### The interplay of host CTL responses and viral evolution in HIV-Nef

In our longitudinal sequence analysis we identified several mutations in and around epitopes targeted by the HLA-A2-restricted CTL responses in subjects OP177 (Nef), OP428 (Gag and Pol), and OP599 (Gag) and next examined the interaction between virus evolution and epitope-specific CTL responses. We first analyzed the interactions between the high-magnitude HLA-A2-restricted AL9 specific CTL response detected at baseline in subject OP177 (Figure 1C), sequence changes and viral load. Our sequence analysis showed that patient OP177 at the earliest sample time point (week 10) carried virus with two different versions of the AL9 epitope, ^83^AAVDLSHFL^91^ [60]. The major population (11/16 clones) was identical to the consensus B sequence (AAVDLSHFL) while the minor population (5/16 clones) contained a L87I substitution (AAVDISHFL) (Table 3). At 13 weeks post-infection, we could detect a potent CTL response against the subtype B consensus epitope sequence, while a less intense CTL response targeted the L87I epitope variant, concurrent with the L87I sequence then dominating the viral population. By week 37 none of 17 clones carried the consensus B sequence and by week 61 the majority of the clones (86%) carried the L87I substitution. Despite a >33 fold increase in viral load, the CTL response against the L87I epitope variant only increased two-fold and the response toward the consensus B specific CTL response decreased substantially indicating that the L87I-specific CTL were less efficient in controlling viral load. After week 61, further mutations were seen within the epitope; some linked with the L87I mutation, others not. In the last sample, drawn at week 158, the major population (13/14 clones) carried two new mutations, A83G and V85L, while the position 87 had reverted back to a wild type leucine, and new, less prevalent HLA-A2-restricted CTL responses towards these variants could be detected.

**Table 3:** **Early viral escape driven by an HLA-A2-restricted response against HIV-Nef.** The interactions between viral evolution (within all defined targeted epitopes), anti-HIV-specific CTL responses, and viral load, was followed in subject OP177.

Week^A^	HIV-Nef Sequence^B^				Clones^C^	CD8+ T cell responses (% IFN-γ and TNF-α)^D^				Viral Load
	HLA-A2	HLA-B40	HLA-A2	HLA-B15						
	AL9	KL9	VL10	WF9						
	^83^AAVDLSHFLKEKGGLEGL^100^		^180^VLEWRFDSRLAF ^191^			AL9	KL9	VL10	WF9	
**10**	**---------**---------		**----------**--		**8/16**					8,805
	**---------**---------		**--------S-**--		2/16					
	**---------**---------		**----------**-L		1/16					
	**----I----**---------		**----------**--		5/16					
**13**	**---------**---------		**----------**--		8/26	**1.10**				17,260
	**----I----**---------		**----------**--		**15/26**	0.14				
	**----I----**---------		**------G---**--		1/26	0.14				
	**----I----**---------		**----------**-C		1/26	0.14				
	**----I----**---------		**----------**-L		1/26	0.14				
**37**	**----I----**---------		**----------**--		8/17					21,600
	**--------I**---------		**----------**--		**9/17**					
**61**	**----I----**---------		**----------**--		**12/14**	**0.31**				295,000
	**----IR---**---------		**----------**--		1/14					
	**--------I**---------		**----------**--		1/14					
**109**	**--L-I----**---------		**----------**--		**7/13**	0.10				164,776
	**--L-I--L-**---------		**----------**--		1/13					
	**--L------**---------		**----------**--		1/13					
	**G-L------**---------		**----------**--		2/13	0.06				
	**G-L------**------D--		**----------**--		1/13	0.06				
	**K-L------**------D--		**----------**--		1/13					
**158**	**--L-I----**---------		**----------**--		1/14	0.13	**1.41**	0.25	0.00	270,035
	**G-L------**---------		**----------**--		**6/14**	**0.19**	1.41	0.25	0.00	
	**G-L------**---------		**----K-----**--		4/14	**0.19**	**1.41**	0.19	1.28	
	**G-L------**------D--		**----------**--		2/14	**0.19**	0.32	0.25	0.00	
	**G-L------**-------E-		**----------**--		1/14	**0.19**		0.25	0.00	

A Estimated week from infection. ^B^ The HXB2 sequence is given as a reference with the HLA-A2 restricted epitopes AL9 and VL10 indicated with an unbroken line in bold face letters, the HLA-B40 restricted epitope KL9 with a dotted line, and the HLA-B15 restricted WF9 epitope with double lines. **^C^** The frequency of each clone is given as a number of the total number of clones sequenced with the major autologous sequence at each time-point given in bold face letters. ^D^The autologous CTL responses are given for each peptide tested. The viral variant that generates the strongest CTL response at each time-point are indicated in bold face letters.

**Figure 4:**
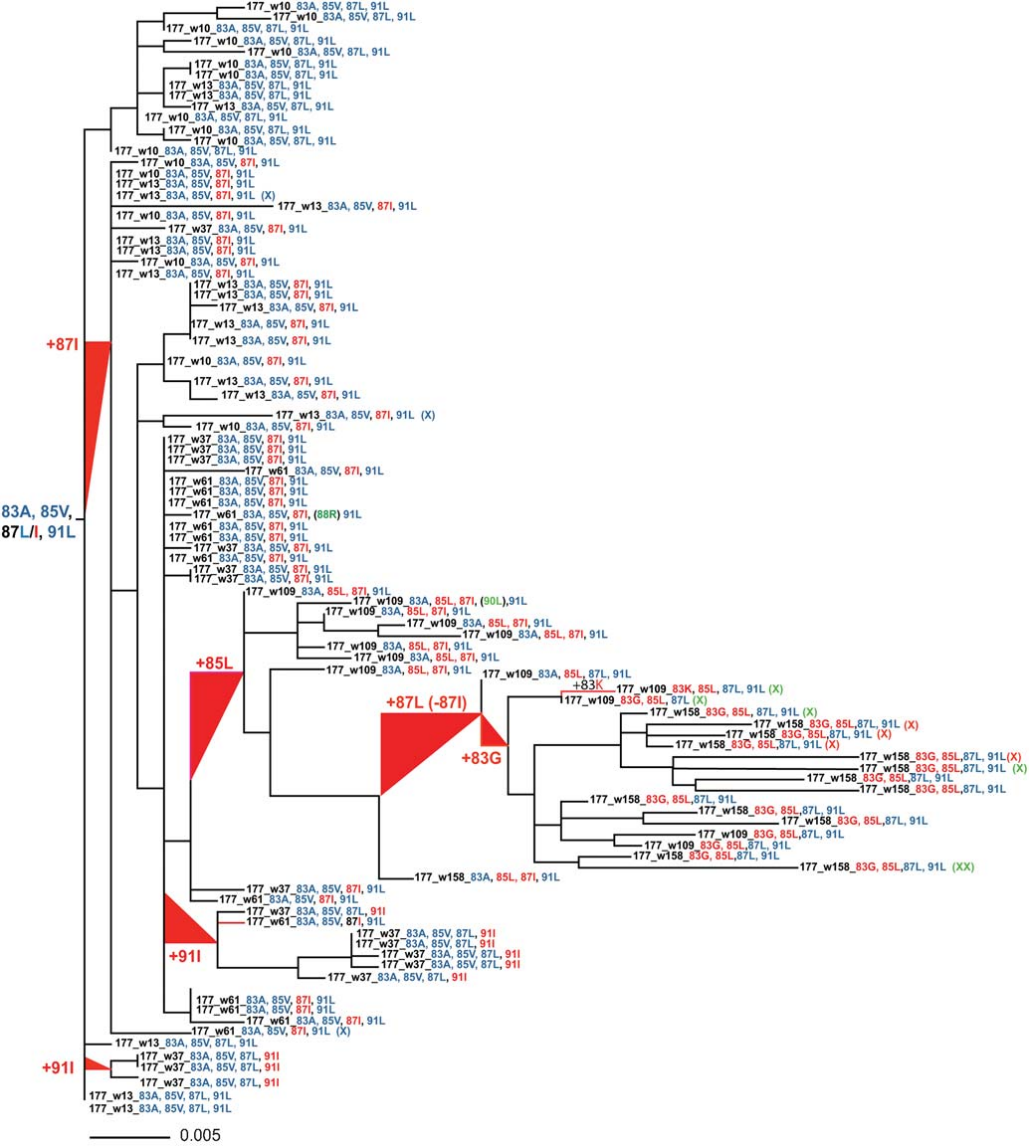
Phylogenetic tree illustrating viral evolution of the HIV-*nef* region (HXB2 coordinates 8774–9540) in subject OP177. Positive selection occurs from the HLA-A2-restricted CTL response (AL9, ^83^AAVDLSHFL^91^) at positions 83, 85, 87 and 91 and the variation at these sites are shown in color on the tree and branches (amino acid positions identical to the consensus B sequence are shown in blue and variations in red) as well as sporadic variation elsewhere in the epitope (shown in green). The patient's virus evolves from the consensus 83A, 85 V, 87L/I, 91L (all blue except 87I which is red) to 83G (red), 85L (red), 87L (blue), 91L (blue) over approximately three years during which various epitope variants with single, or linked mutations occur in the population (83K, 87I, 91I, shown in red). Of note is the temporary predominance of the 87I mutation, which is lost shortly after the 85L mutation develop. An X at the end of the clone name refers to changes in other epitopes; green X = KL9 epitope, E98D, green XX = KL9 epitope, G99E, red X = VL9 epitope and overlapping WF9 epitope, R185K, blue X = sporadic changes in WF9 epitope (Table 3). Amino acid numbering corresponds to HXB2 Gag. Scale bars signify substitutions/site. w: corresponds to estimated week from infection (e.g. a clone named 177_w13 are obtained from a plasma sample drawn at week 13).

To evaluate the impact of these epitope changes on the evolution of the viral population in OP177 we superimposed the changes on a maximum-likelihood phylogenetic tree created using the full-length *nef* sequences (Figure 4). Furthermore, using an independent phylogenetic method, codeml [49], we identified 20 amino acid positions undergoing positive selection within the Nef region. Nine of the positions (22, 50, 64, 83, 85, 87, 91, 98, and 184) fell within known CTL epitopes, restricted by the patients HLA alleles (A2, A3, B15, and B40). These analyses confirmed that several of the amino acid positions in the AL9 epitope (83, 85, 87 and 91), were under positive selective pressure, presumably caused by the HLA-A2-restricted CTL response (Figure 4). To evaluate the potential contribution of additional CTL responses against the region we tested all the appropriate autologous peptide variants of eight known CTL epitopes, and also included six peptides from the HIV-1 group M consensus Nef (15-mer) peptides set (NIH AIDS Research and Reference Program, Division of AIDS, NIAID, NIH) spanning five other positions, in a sample obtained at estimated week 158 from infection. CTL responses were identified against three epitopes, containing positions E98D and R184K (Table 3). An HLA-B40 restricted response was detected against the Nef 92-100 (^92^KEKGGLEGL^100^, KL9) epitope, adjacent to the HLA-A2 restricted AL9 epitope. A high frequency (1.41%) of the CD8 T-cells targeted the wild-type variant and only a minority (0.32%) were against the emerging E98D containing variant, which was found at low frequency in the clones from weeks 109 and 158. Overlapping HLA-A2 (^180^VLEWRFDSRL^189^, VL10) and HLA-B15 (Nef 183-191, ^183^WRFDSRLAF^191^, WF9) restricted CTL responses differentially recognized the 184K mutant. The HLA-A2-restricted VL10 primarily recognized the wild-type version of the epitope while a potent HLA-B15 restricted response was directed against the emerging 184K variant of the epitope (1.28%), but did not recognize the wild-type version. In summary, we could confirm that the adjacent positions 83, 85, 87, 91, and 98 all were under pressure by flanking HLA-A2 and HLA-B40 restricted CTL responses and that position 184 fell under two overlapping CTL responses restricted by HLA-A2 and HLA-B15. Also, early escape from the AL9 CTL response occurred simultaneously with a sharp increase in viremia. Although we found a clear association between CTL responses and viral evolution for six of the positions under positive selection, no such association was found for the other 14 positions. As we were unable to obtain and test a sample drawn earlier than week 158 we cannot exclude the presence of additional CTL responses at earlier time-points. However, the majority of the sequence changes were seen at the later time-points, in clones obtained at week 109 and 158, so it is unlikely that we would have failed to detect the presence of responses in our sample from week 158. This notion is supported by the fact that we could still detect HLA-A2-restricted CTL responses against the initial wild-type AL9 epitope (0.20% IFN-γ producing CD8+ T cells), a sequence that had not been detected since week 13. Instead, as several of the amino acid changes (50, 133, 174, 176) were associated with reversion toward a consensus-B like viral sequence, it is possible that many amino-acid changes throughout the Nef sequence were selected for because they conferred an increase in viral fitness.

### Viral adaptation induced by HLA-A2-restricted Nef-specific CTL

Patient OP177 was one of two HLA-A2-positive patients studied who had a virus identical to the consensus B sequence within the AL9 epitope in early infection, and the only one who developed CTL responses against the epitope (Table 4). Baseline virus sequences from the other eight HLA-A2-positive patients all carried glycine in position 83, which in seven of the patients was linked to a leucine, phenylalanine or arginine in position 85 (Table 4). To evaluate if the changes could be due to very early CTL escape not detected in our baseline samples, we obtained viral sequences from twelve matched HLA-A2-negative subjects identified at primary infection (Table 1). Similar to the HLA-A2-positive subjects, eleven of these twelve had a glycine in position 83 and/or leucine or phenylalanine in position 85 (Table 4). When compared to sequences obtained after a mean of 96 weeks of follow-up, none of HLA-A2-positive (except OP177) or HLA-A2-negative patients showed any sign of mutations or reversions within the epitope except for patient OP478 who was identified as being superinfected with a second viral variant during the first year of infection (F.M.H., unpublished data). None of the HLA-A2-positive subjects who acquired a variant of the epitope at primary HIV-1 infection were able to generate a CTL response against autologous virus during the study period. Furthermore, in the absence of an autologous Nef-specific CTL response, the mutations persisted in both HLA-A2-positive and HLA-A2-negative subjects for years. Our results suggest that the selective pressure induced by an HLA-A2-restricted early CTL response towards the consensus B version of the AL9 epitope has induced a change in the epitope that has become fixed in the majority of circulating HIV-1 subtype B viruses, possibly because no, or only negligible, fitness cost is associated with the change. This is a novel example of viral adaptation to an HLA-A2-restricted CTL response.

**Table 4:** Signature pattern and viral evolution within the HLA-A2-restricted AL9 epitope.

Subject	Epitope Sequence	
	Baseline	Follow up^A^
	AAVDLSHFL^B^	AAVDLSHFL^B^
HLA-A2-Positive		
OP177	---------	G-L------
OP428	---------C	---------
OP474	G--------	n.a.
OP478	G--------	--R------D
OP454	n.a.	G-L------
OP488	G-L------	G-L------
OP506	G-L------	n.a.
OP539	G-L------	G-L------
OP581	G-L------	G-L------
OP583	G-R------	G-R------
OP599	G-L------	G-L------
HLA-A2-Negative		
OP138	G-L------	G-L------
OP430	G-L------	G-L------
OP443	--I------	--I------
OP470	G-L------	G-L------
OP565	---------	---------
OP626	G-L------	G-L------
OP683	G-F---F--	G-F---F--
OP700	G-L------	G-L------
OP722	G-L------	G-L------
OP745	G-L------	G-L------
OP791	--L------	--L------
OP842	--L------	n.a.

A The follow up sequence was available from a mean of 96 weeks from base-line (range: 72–144 weeks). ^B^The Consensus B sequence of the AL9 epitope is given as a reference for amino acid position 83-91. **^C^**Sequence obtained at estimated week 39 from infection. ^D^Viral evolution due to a confirmed case of HIV superinfection within the first year of infection (F.M.H., unpublished data). Dashes represent identity with the reference sequence.

**Table 5:** Viral escape from an intricate HIV-Gag specific CTL response restricted by three HLA-alleles followed in patient OP428.

Week^A^	HIV-Gag p17 Sequence^B^			Clones^C^	CD8+ T cell responses (% IFN-γ and TNF-α)^D^			Viral Load (copies/ml)
	HLA-A1	HLA-A2	HLA-B8					
	GY9	SL9	EL9					
	^71^GSEEL RSLYNTVATL YCVHQRIEVK DTKEAL ^101^				SL9	GY9	EL9	
**15**	----- -**-----I---** ---------- ------			**15/16**	0.00			2,340
	----- -**-----I---** ---------- ---G--			1/16	0.00			
**39**	----- -**-----I---** --------I- ------			**9/14**	**0.32**			37,628
	----- -**-----I---** -----G---- ------			4/14	**0.32**.			
	----- -**----AI---** -----G---- ------			1/14				
**63**	----- -**-----I---** -----G---- ------			**5/12**	**1.17**	**0.80**	0.04	122,968
	----- -**-----I---** -------D-- ------			3/12	**1.17**	**0.80**	**0.80**	
	----- -**--F--I---** -------D-- ------			1/12	0.63	0.04	**0.80**	
	----- -**--F--I---** -----G---- ------			2/12	0.63	0.04	0.04	
	----- -**--C--I---** -----G---- ------			1/12				
**88**	----- -**-----I---** -----G---- ------			**8/14**	**0.77**			190,263
	----- -**--F--I---** -----G---- ------			6/14	0.64			
**139**	----- -**-----I---** -----G---- ------			2/11				256,432
	----- -**--F--I---** -----G---- ------			**8/11**				
	----- -**--F--I---** -----G-K-- ------			1/11				
**156**	----- -**-----I---** -----G---- ------			1/14	**0.77**			290,576
	----- -**--F--I---** -----G---- ------			**13/14**	0.53			

A Estimated weeks from infection. ^B^The Consensus B sequence is given as a reference for amino acid position 71 to 101 of the HIV-1 Gag p17 region. The HLA-A2 restricted epitope SL9 is underlined with a single unbroken line, the HLA-A1 restricted epitope GY9 with a dotted line, and the HLA-B8 restricted epitope EL9 with double lines. **^C^**The frequency of each clone is given as a number of the total number of clones sequenced. The major autologous sequence at each time-point is given in bold face letters. ^D^The autologous CTL responses are given for each peptide tested. The at week 63 minor viral sequence, which by week 139 becomes the major viral sequence, are indicated by a black box. Likewise, the corresponding CTL responses are surrounded by black boxes for each of the HLA-A2-, HLA-A1-, and HLA-B8-restriced responses, respectively. The viral variant that generates the strongest CD8+ T cell response for every HLA-restriction at each time-point is given in bold.

### HLA-A1 and -A2-restricted CTL responses select for dual escape mutation

**Figure 5:**
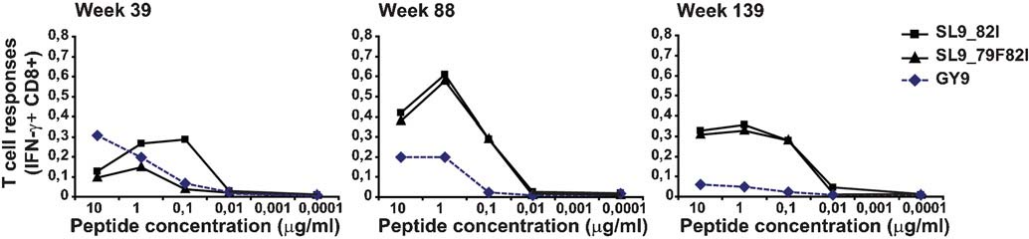
Characterization of the HLA-A2-restricted SL9-specific (82I and 79F82I autologous variants) and HLA-A1-restricted GY9 specific CTL responses in OP428. Peptide titration of the autologous epitope variants was conducted to evaluate the functional avidity of the responses over time, at weeks 39, 88, and 139 from estimated infection. The CFC assay was used to measure the production of gamma interferon by antigen specific CD8+ T cells, with the indicated peptides in 10-fold dilutions.

**Figure 6:**
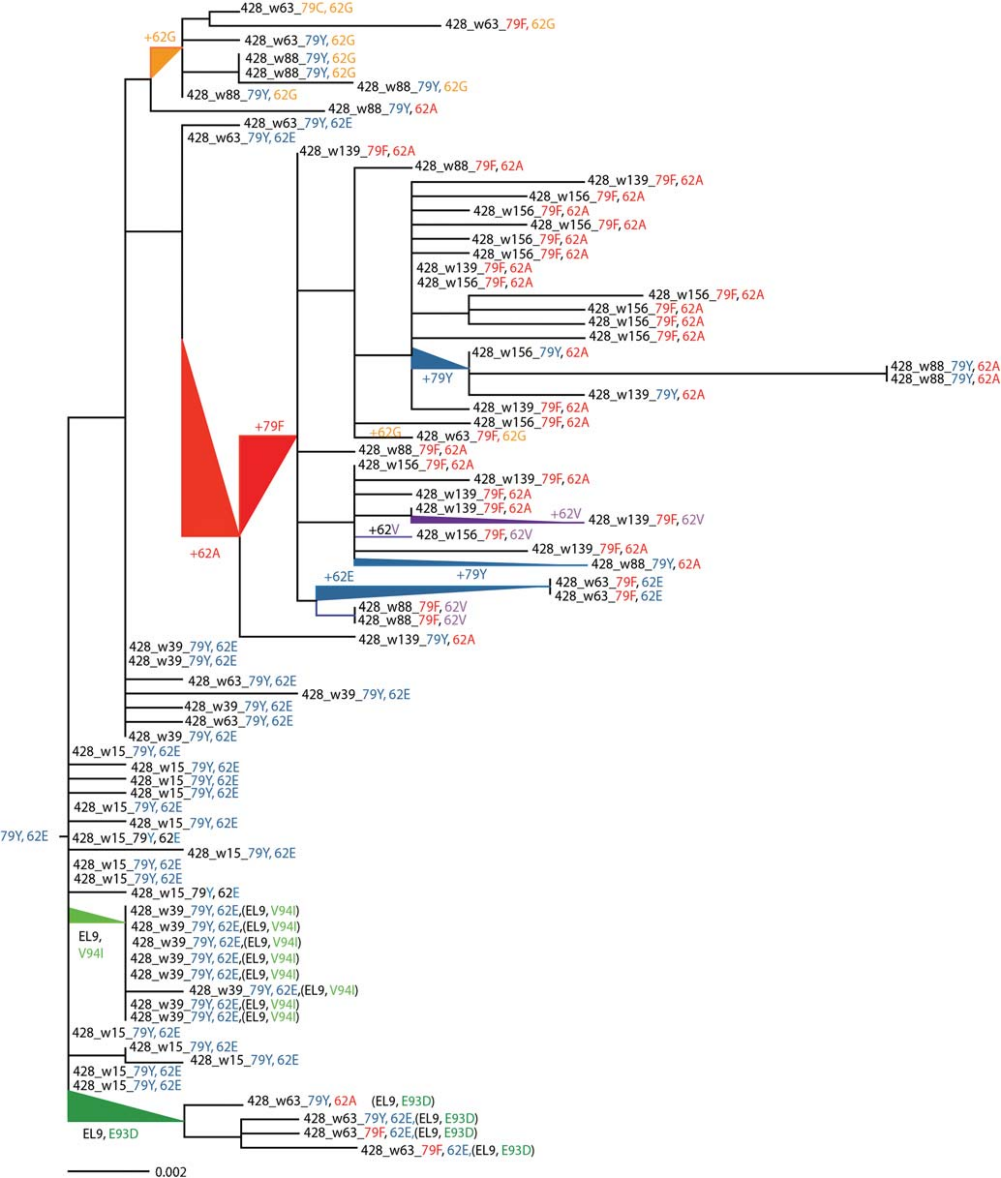
Phylogenetic tree illustrating viral evolution of the HIV-p17 *gag* region (HXB2 coordinates 790–1431) in subject OP428 under selective pressure. Positively selected amino acid sites within the HLA-A2 ((SL9, ^77^SLYNTIATL^85^ (patient consensus)), HLA-A1 (GY9, ^71^GSEELRSLY^79^), and HLA-B8 (EL9, ^93^EVKDTKEAL^101^) restricted epitopes. Amino acids corresponding to the consensus B sequence are shown in blue, while the Y79F mutation within SL9 and GY9 is given in red, and the E93D and V94I mutations within EL9 are shown in green on the tree. The variation found at the positively selected amino acid position 62, where a potential compensatory mutation (E62A) seems to occur prior to, and associated with, the Y79F substitution are as follows: variation at position 62: E, blue, G, orange, V purple and A red. Amino acid numbering corresponds to HXB2 Gag. Scale bars signify substitutions/site. w: corresponds to estimated week from infection (e.g. a clone named 428_w15 was obtained from a plasma sample drawn at week 15).

Patient OP428, the third patient who developed a broad HLA-A2-restricted response during early chronic infection, also had an intricate interaction between cellular immune responses restricted by several HLA-alleles and viral evolution (Table 5). We detected an HLA-A2-restricted CTL response against the major autologous, Gag SL9 epitope variant ^77^SLYNTIATL^85^ (82I) by week 39, and by week 63 several clones had developed an additional Y79F mutation in position three of the epitope (^77^SLFNTIATL^85^). We measured the cellular immune response against the two viral variants and found the 79F mutant to elicit a 50% lower magnitude and a log lower functional avidity CTL response than the initial V82I variant (Figure 5). By week 88, when 42% of the clones contained the 79F mutation, the magnitude and functional avidity of CTL recognition of the 79F82I variant had increased and was similar to the recognition of the initial SL9-82I variant indicating sequential maturation of the mutant-specific response (Figure 5). Still, by weeks 139 and 156 the vast majority of the viral clones contained the Y79F mutation. Phylogenetic analysis showed positive selection of the Y79F substitution as well as an E62G/V/A change upstream of the SL9 epitope. When we analyzed the viral population in the context of the phylogenetic tree (Figure 6), the Y79F mutant was seen in combination with both 62E and 62G at week 63 from infection. However, this combination did not propagate very successfully and at later time points the virus population primarily carried a combination of the 62A and 79F mutations, coinciding with substantial increases in viral load (Table 5). Structural analysis of the p17 monomer showed that the side-chain of the amino acid in position 62 interacts with the side-chain of the amino acid in position 79 (A.K.N.I., unpublished data, and [61]). The 62A mutation may thus be a compensatory mutation reducing, or eliminating, any fitness cost associated with the Y79F escape mutation and the potential immunologic impact imposed by the developing HLA-A2-restricted CTL response against the 79F variant epitope. HIV-1 subtype B virus rarely carries an alanine in position 62 (<1%), but the frequency is increased in combination with 79F (5%) [62].

Additional amino acid changes were detected close to positions Y79F and V82I in this epitope rich region of p17 Gag. We compared defined epitopes with OP428's HLA type (A1, A2 and B8), and found three additional epitopes to which the patient could respond. One was an HLA-A1 restricted epitope spanning Gag 71-79 (GY9), and two were B8 restricted epitopes spanning Gag 74-82 (EV9) and Gag 93-101 (EL9). While we detected no CTL response against the EV9 epitope, responses could be found against both the GY9 and EL9 epitope at week 63 (Table 5). Approximately 0.8% of the patients CTLs targeted the GY index epitope, ^71^GSEELRSLY^79^, while there was no recognition of the Y79F variant epitope. Neither did the patient develop any response targeting the Y79F variant epitope as measured in samples obtained at weeks 88 and 139 (data not shown). The 79F substitution is situated in the anchor position of the HLA-A1-restricted epitope, and most likely negatively affects binding to the HLA molecule. In comparison to the HLA-A2-restricted CTL responses against the autologous SL9 epitope, the functional avidity of the HLA-A1-restricted GY9 response was lower. Also, by week 139, when the majority of the viral clones contained the 79F mutation, the magnitude of the response was on the verge of becoming undetectable indicating that there was no cross-recognition of the 79F mutant by the GY9-specific CTL response. Thus, the 79F substitution affects CTL responses against both the SL9 and especially the GY9 epitopes, and seems to be a potent CTL escape mutation. Strong positive selection of 79F was detected using phylogenetic analysis (codeml from PAML) and supported our functional data [49]. In OP428 the viral load increased substantially during the period in which the viral population successfully evaded anti-Gag-specific CTL-responses restricted by HLA-A1, and A2 and developed a potential compensatory mutation in position 62 (Table 5, Figure 6).

### Selection of the subtype-B consensus sequence induced by CTLs recognizing mutant epitope variants

With regard to the responses seen in patient OP428 targeting the HLA-B8 restricted EL9 epitope (Table 5), we found that 0.8% of the CTLs were actually directed at the emerging E93D mutant epitope (^93^ DVKDTKEAL^101^) at week 63, while there was no recognition of the index sequence, ^93^EVKDTKEAL^101^. The E93D mutation was only detected in the viral sequences from this time point and our phylogenetic tree analysis showed that all the clones, which carried the E93D mutation, clustered together on a single branch of the tree and did not spread any further, demonstrating the effectiveness of the CTL response in eliminating cells infected with these viral variants (Figure 6). An additional mutation, R91G, is seen upstream of the EL9 epitope and downstream of the SL9 epitope. While there are no epitopes described overlapping this position, that OP428 could recognize, the mutation may affect processing of the EL9 epitope. In OP428 the viral load increased substantially during the period in which the viral population successfully evaded anti-Gag-specific CTL-responses restricted by HLA-A1, A2, and B8 (Table 5).

In addition to the multiple targeting of the HIV-Gag p17 region, subject OP428 had a CTL response towards an HLA-A2-restricted epitope in the RT region of HIV-Pol, RT 33-41 (Table 6). Initially, the major viral population carried an isoleucine in position three of the epitope, ^33^ALIEICTEM^41 ^(35I), while no subtype B consensus epitope sequences were found. By week 63, a new variant, ^33^ALMEICTEM^41^ (35M), started to emerge and became the major viral sequence by week 88. The 35M substitution did not persist and by week 156 was replaced by a virus identical to the consensus B sequence ^33^ALVEICTEM^41^, an example of evolution of the transmitted virus variant toward the subtype B consensus sequence.

**Table 6:** An emerging anti-HIV-Pol mutant-specific CTL response responsible for selection of the consensus B like sequence in patient OP428.

Week^A^	HIV-Pol RT Sequence^B^	Clones^C^	CD8+ T cell responses (% IFN-γ and TNF-α)^D^
	^28^EEKIKALVEICTEMEKEGK^46^		
**39**	-----**--I------**-----	**15/17**	0.00
	-----**--T------**-----	1/17	n.a.
	-----**--I-----T**-----	1/17	n.a.
**63**	-----**--I------**-----	**13/14**	n.a.
	-----**--M------**-----	1/14	n.a.
**88**	-----**--I------**-----	4/12	0.15
	-----**--I----D-**-----	1/12	n.a.
	-----**--M------**-----	**5/12**	**0.34**
	-----**--MK-----**-----	1/12	n.a.
	-----**---------**-----	1/12	0.01
**156**	-----**--I------**-----	1/13	0.47
	-----**--M------**-----	3/13	**0.56**
	-----**--M---A--**-----	1/13	n.a.
	-----**---------**-----	**8/13**	0.14

A Estimated weeks from infection. ^B^The consensus B sequence spanning amino acid 28 to 46 in RT is given as a reference with the epitope RT 33-41 given in bold face letters. **^C^**The frequency of each clone is given as a number of the total number of clones sequenced. The major autologous sequence at each time-point is indicated in bold face numbers. ^D^The autologous CTL responses are given for each peptide tested. The viral variant that generates the strongest CTL response at each time-point are given in bold.

We went on to study what impact the cellular immune response had on the evolving viral population and determined the CTL response against the subtype B consensus sequence and the two patient variants, 35I and 35M at week 88 (Table 6). We found that the main target was the 35M variant, which was the most frequent epitope sequence found in the viral population, followed by a weaker response to the 35I variant, which dominated the population at weeks 39 and 63. We could only detect negligible CTL responses directed at the emerging subtype B consensus epitope. At week 156, the major CTL targets continued to be the two mutant epitopes, despite the consensus B epitope sequence being the major viral variant. The phylogenetic analysis showed strong positive selection at position 35 as the epitope changed from I to M to V (AKNI, unpublished data). Together with the CTL response detected against the EL9 epitope in HIV-Gag, this is our second example of how HIV-1-specific CTL responses directed at variant epitope sequences can cause the virus epitope to develop into a consensus B-like sequence.

### Increasing magnitudes of HIV-Gag restricted CTL responses associated with broad CTL responses and viral escape

**Figure 7:**
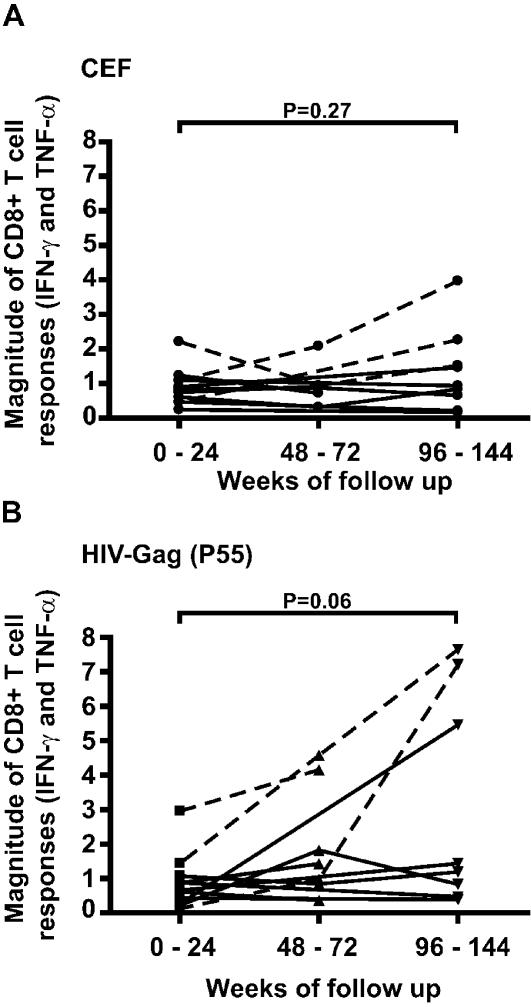
Increase of HIV-Gag responses over time. The cellular immune responses against the control peptide pools CEF (A) and HIV-Gag (B) were followed in longitudinally drawn PBMC samples. No statistically significant differences in CTL responses to non-HIV antigens included in the CEF peptide pool were seen over time (Paired T-test, p = 0.269). However, the increase in CTL response to the HIV-Gag (p55) peptide pool was borderline significant (Paired T-test, p = 0.064). The responses in subjects OP177, OP428 and OP599, who developed multiple HLA-A2-restricted CTL responses and showed evidence of viral escape within targeted epitopes, are indicated with dashed lines.

We next evaluated the longitudinal cellular immune responses against two control antigens, a peptide pool consisting of well-identified HLA class I restricted CMV, EBV and Influenza epitopes (CEF), and a peptide pool consisting of overlapping 11-mer peptides spanning the whole HIV-Gag p55 region (Figure 7). During the study period the overall magnitude of the CTL responses towards the CEF peptide pool remained stable (Figure 7A) while the cellular immune responses against HIV-Gag increased, especially in three patients, OP177 and OP428 and OP583 (Figure 7B). Subjects OP177 and OP428 were also the only subjects who showed an increase in response against the CEF peptide pool. Both OP177 and OP428 developed multiple HLA-A2-restricted CTL responses and showed evidence of viral escape within targeted epitopes, while OP583 had no detectable HLA-A2-restricted responses. In OP177 and OP428 the increase in magnitude of CTL responses against HIV-Gag were associated with an increased viral load during the first two years, as these patients had a viral load increase of 1.27 and 1.66 log_10_, respectively, compared to 0.31 log_10_ in OP583. A fourth patient, OP599, had a high magnitude of HIV-Gag CTL responses at week 72, while targeting multiple HLA-A2-restricted epitopes, and these epitopes (including SL9) showed signs of viral evolution.

## Discussion

We have identified different modes of viral adaptation in response to dynamic fluctuating inter and intra epitopic CTL responses following primary HIV-1 infection. The continuously developing CTL responses were detected both against an unchanged viral population forcing viral evolution to the point of HLA-A2 adaptation, as well as against existing mutants inducing reselection of a consensus B like viral variant. Broad CTL responses were directed against epitopes in Gag, Pol, Env, and Nef and developed in 27% of our patients during the first year of infection and were associated with the induction of viral escape mutations in targeted epitopes. We confirmed in two of three subjects with broad responses that overlapping and flanking CTL responses of different HLA-restrictions were simultaneously targeting the HIV-Gag or HIV-Nef region. Surprisingly, while the three subjects with an early broadening of HLA-restricted HIV-1-specific CTL responses experienced an increase in viral plasma load during the first year of infection, the vast majority of our patients (6/7) who either had no HLA-A2-restricted CTL responses, or responses towards Gag only (n = 2), had stable plasma RNA levels during this period. While the viral load increased in the group with broad HLA-A2-restricted CTL responses, after year one the average viral loads were similar. This suggests that at least some of the time, broadening CTL responses can be associated with escape from earlier temporarily effective immune responses, and can be a footprint left by effective viral evasion of CTL responses rather than demonstrating immune control.

In chronic untreated infection, 70-75% of all subjects recognize the subtype B consensus SL9 sequence [55], [56], [58] and >90% recognize one or more of the common variants [61], suggesting that the development of responses against this epitope, and epitope variants, is a process that continues beyond the first years of infection, as only 6/11 recognized this epitope after the first year. The development of CTL responses was consistently associated with the induction of viral escape mutations in the targeted epitopes, which was further linked to the development of novel CTL responses towards the new epitope variants. This finding supports the concept of antigenic oscillations and shifting immunodominance in HIV-1 infection described earlier, based on phylogenetic analysis of viral sequences [63]. New CTL responses developed towards epitopes, which had remained unchanged since primary infection, suggesting that sequential maturation of the cellular immune responses occurs throughout this period.

CTL responses towards all HIV-1 genes have generally been thought to have an inhibitory effect on disease progression, although responses restricted by certain HLA molecules, e.g. B27, B57, and B58, are thought to be more efficient than others in constraining viral replication. Our data, which is one of the most comprehensive studies of a particular set of HLA-restricted CTL responses and viral evolution to date, suggest that developing broad responses against epitopes in Gag, Pol, Env, and Nef, in association with the development of escape mutations, have a negligible effect on viral load. As we studied the major HIV gene sequences, it is possible that there were CTL responses to other parts of the virus that were not studied and also influenced viral load. Our observation is supported by data from a study of CTL responses in a cohort of long-term non-progressors (LTNP) in whom broad CTL responses were associated with a median viral load 30 times higher than that found in patients with no CTL responses and loss of LTNP status within 4 years of study entry [64]. However, the extended detailed analysis of the impact the overall CTL responses had on viral evolution in one patient in early chronic infection (week 158 from primary infection) showed that several of the amino acid positions under positive selection in HIV-Nef were not recognized by CTL. Instead, several of these mutations were associated with reversion towards consensus B like variants, supporting the notion that some of these mutations may lower viral fitness [21], [33], [34], [35]. Although we did not directly measure viral fitness, and thus the fitness cost of the CTL escape mutations that we identified can not be fully characterized, our findings suggest that mutations introduced to enhance viral fitness may compensate for the potential effect developing CTL responses may have on viral load, pointing out the importance of understanding these complex interactions to be able to identify which CTL responses are most advantageous.

The HIV-Nef region has previously been shown to be preferentially targeted in primary HIV-1 infection [65], [66]. However, we found early HLA-A2-restricted anti-Nef-specific CTL responses in only one of our patients, OP177. Over time, the viral population in this patient developed mutations in the targeted AL9 epitope at amino acid positions 83, 85, 87 and 91. These mutations occurred both alone, and linked, and after three years the dominant epitope variant (83G, 85L) was similar to that most frequently found in both HLA-A2-positive and negative patients in our study as well as in the database. Thus, in other patients, the CTL escape mutations we observed develop in OP177, which may have been transmitted, and persisted after transmission, suggesting a negligible fitness cost. In the Caucasian population, the HLA-A2 allele is the most prevalent HLA allele, present in around 46% of all individuals [67]. It is thus possible that the selective pressure induced by the HLA-A2-restricted Nef CTL response over time has been shaping the circulating viral population resulting in the 83G–85L variant being fixed at the population level. As this epitope has been shown to be targeted by CTL responses restricted to additional HLA-alleles, HLA-B60 and Cw*03 [68], these responses may have contributed to shaping of the viral population. Transmission and accumulation of CTL escape variants has previously only been shown in HLA-B57/B5801 positive individuals [21], and epitope variants selected in this patient group may take longer to accumulate in the viral population due to the lower prevalence of HLA-B57 relative to HLA-A2.

HIV p17 Gag contains many regions of overlapping CTL epitopes restricted by multiple HLA molecules, including the one carrying the HLA-A1 and HLA-A2-restricted CTL epitopes, GY9 (^71^GSEELRSLY^79^) and SL9 (^77^SLYNTVATL^85^). In one patient, OP428, we found a dynamic interaction and synergy between the CTL responses towards these two epitopes and viral evolution. The cumulative responses were responsible for the positive selection of the dual CTL escape virus variant carrying an Y79F substitution. The 79F mutation initially reduced the response to SL9 by approximately 50%, and abolished recognition of GY9. As the 79F mutation is located in the anchor position of the HLA-A1-restriced epitope, our finding suggests that the 79F mutation prohibited binding of GY9 to the HLA-A1 molecule. For the HLA-A2-restricted SL9 epitope, it was recently described that the Y79F and V82I mutations affect T cell-receptor contact [61]. While Iversen *et al*. found that most chronically infected, HLA-A2-positive patients with evidence of positive selection for escape mutations in SL9, or fixed escape variants, had lower viral loads than patients carrying the wild type sequence, we found that escape mutations within the overlapping GY9 and SL9 epitopes in our patient in early infection correlated with an increase in viral load. The difference between our patient and the general trend may be due to the positive selection of alanine at position 62, which in combination with the Y79F substitution, propagates very successfully and dominates the viral population at later time points. Structural analysis suggested that the side-chain of the amino acid in position 62 interacts with that of the amino acid in position 79, and thus may be a compensatory change reducing, or eliminating, any fitness cost associated with the escape mutation in SL9. This is supported by the observation that the combination of 62A and 79F is more than five times as common as 62A alone among the subtype B virus variants in the HIV database (30). The added viral advantage of escaping both an HLA-A1 and HLA-A2-restricted immune response may also lead to higher viral loads. It is likely, that although the changes in our patient conferred immune evasion, another host may have highly efficient T-cells able to target the viral variants [61], [69], [70]. In our subject we identified development of a high avidity CTL response against the 79F variant epitope after the initial viral load increase. Our results demonstrate the role CTL responses play in shaping viral evolution, once more pointing out the importance of understanding the interaction between selective forces induced by CTL and functional and structural constraints of the virus.

We found two independent examples of potent CTL responses generated against epitope variants, which resulted in selection of the subtype B consensus sequence. In the first case an HLA-B8-restricted CTL response was generated against an emerging mutant epitope in the HIV p17 Gag region and in the second, HLA-A2-restricted CTL responses targeted sequential epitope variants in reverse trancriptase of HIV-Pol. Although the generation of de novo escape-variant-specific CTL responses has been described in chronic HIV-1 infection [71], this is the first example of rapid development of potent responses in early infection. While the SL9-specific CTL responses arose against epitope sequences that had remained constant since primary infection, other responses can develop against a changing viral population.

Our data is in line with the notion of HLA adaptation [29], [31]. It is thought that viral evolution is the result of a trade-off between the benefit of escaping CTL responses and the impact of CTL escape mutations on viral fitness [69]. Efficient variant-specific CTL responses to several epitopes restricted by HLA-A2, HLA-B8, and HLA-B15 in our study indicate that some very common epitope sequences previously have been adapted by a potent CTL response. This notion is supported by the fact that after sexual transmission, viral CTL escape mutations may be retained [72] and reversion of the mutations can be very slow, or non-existent [21]. Thus, selection and preservation of certain amino acids within CTL epitopes in the HIV-genome may not only be dependent on functional or structural constraints but also on how well they are recognized by effective CTL responses.

Our data reinforce the concept that not all CTL responses are equally beneficial to the patients and some represent immunological footprints of earlier effective immune responses from which the virus has escaped. Our results showed a greater increase in viral plasma load during the first year of infection in subjects who developed early broad HLA-restricted responses in association with viral escape than in subjects with no or narrow HLA-A2- responses towards the SL9 epitope in p17 Gag only. Thus, therapeutic vaccination with different CTL epitope antigens should only target epitopes clearly associated with containment of viral load or possibly in areas where CTL escape mutations are associated with substantial viral fitness costs.

## Supporting Information

Table S1 Primer sequences
(0.03 MB DOC)
