# Supplementary material for: Sequential Broadening of CTL Responses in Early HIV-1 Infection Is Associated with Viral Escape
Source: PLoS One. 2007 Feb 21;2(2):e225. doi: 10.1371/journal.pone.0000225 (PMC1790860; doi:10.1371/journal.pone.0000225)
Supplement: Table S1 — Primer sequences (0.03 MB DOC) [file pone.0000225.s001.doc]

### Table S1. Primer sequences

| Region | **Forward 5’-Primer** | **HXB2 nt position^A^** | **Reverse 3’-Primer** | **HXB2 nt position** |
| --- | --- | --- | --- | --- |
| **Gag_Out** | GCG GAG GCT AGA AGG AGA GAG | **768-789** | TGC TGT CAT CAT TTC TTC TAR TGT | **1836-1813** |
| **Gag_In / Seq^B^** | ATG GGT GCG AGA GCG TCA GTA T | **790-811** | TCT ATC CCA TTC TGC AGC TTC | **1431-1411** |
|  |  |  |  |  |
| **Nef_Out** | TaA GAC AgG Gct TgG AAA gR | **8764-8783** | ctc aag gca agc ttt att gag gc | **9629-9607** |
| **Nef_In / Seq** | Cca gta cag gca aaa agc ag | **9521-9540** | ctT gGA AAg ggc tTT gct aTA A | **8774-8795** |
|  |  |  |  |  |
| **Pol_Out** | TTG GAA ATG TGG AAA GGA AGG AC | **2028-2050** | CTG TAT TTC TGC TAT TAA GTC TTT TGA TGG G | **3539-3509** |
| **Pol_In** | CAG AGC CAA CAG CCC CAC CA | **2147-2166** | CTG CCA GTT CTA GCT CTG CTT C | **3462-3441** |
| **Pol_Seq** | AGC AGG AGC MGA WAG ACA RGG | **2213-2233** | TGG TGT YTC ATT RTT TRY ACT AG | **2969-2947** |
|  |  |  | TTA ATC CCT GCR TAA ATC TGA CTT G | **3373-3349** |

^A^Nucleotide positions correspond to the HXB2 reference sequence. ^B^ Primer used for sequencing.
